# Clinical Ethics Consultation in Chronic Illness: Challenging Epistemic Injustice Through Epistemic Modesty

**DOI:** 10.1007/s10730-022-09494-8

**Published:** 2022-09-07

**Authors:** Tatjana Weidmann-Hügle, Settimio Monteverde

**Affiliations:** 1 Institute of Biomedical Ethics and History of Medicine, University of Zurich, Zurich, Switzerland; 2 Clinical Ethics Unit, University Hospital Zurich, Zurich, Switzerland; 3 School of Health Professions Bern, University of Applied Sciences, Bern, Switzerland

**Keywords:** Clinical ethics, Clinical ethics consultation, Chronic illness, Epistemic injustice, Biomedical model of disease, Scientific epistemology

## Abstract

Leading paradigms of clinical ethics consultation closely follow a biomedical model of care. In this paper, we present a theoretical reflection on the underlying biomedical model of disease, how it shaped clinical practices and patterns of ethical deliberation within these practices, and the repercussions it has on clinical ethics consultations for patients with chronic illness. We contend that this model, despite its important contribution to capturing the ethical issues of day-to-day clinical ethics deliberation, might not be sufficient for patients presenting with chronic illnesses and navigating as “lay experts” of their medical condition(s) through the health care system. Not fully considering the sources of personal knowledge and expertise may lead to epistemic injustice within an ethical deliberation logic narrowly relying on a biomedical model of disease. In caring “for” and collaboratively “with” this patient population, we answer the threat of epistemic injustice with epistemic modesty and humility. We will propose ideas about how clinical ethics could contribute to an expansion of the biomedical model of care, so that important aspects of chronic illness experience would flow into clinical-ethical decision-making.

## Introduction

Since the middle of the last century, medical progress in understanding disease mechanisms and discoveries about how to cure or influence the course of illness have helped to establish the biomedical model of disease as the predominant approach of clinical decision making, resulting in a biomedical model of care. In a biomedical conceptualization, disease is primarily understood as a natural scientific phenomenon (Lenz, 2018; Stein, 2012; Sulik, 2011). Accordingly, in a biomedical conceptualization of care, the focus of treatment is a pathological condition, a biophysical flaw, that is viewed as a quantitative deviation from the normal state of health. Little attention is thereby paid to the immediate experience of illness, the plethora of impacts on a person’s life that the illness entails, and the way in which an affected person integrates illness into her life (Carel, 2008; Frank, 2002; Toombs, 1992). In addition, health care systems in wealthy countries have evolved in ways that have emphasized the treatment of acute disease events. Conceptually and structurally, modern healthcare systems rest upon an episodic, acute medical model of care. Likewise, biomedical and clinical ethics has also focused on acute and emergency care. Problems encountered in the context of chronic disease have received little attention (Gibson & Upshur, 2012).

Clinical ethics consultation (CEC) as well as structures and methods for ethical decision-making arose within and attend to crises in the acute-care hospitals. Acute care hospitals are characterized by high labor division, highly structured workflow, the availability of high-technology infrastructure, and a wide range of specialists—including clinical ethicists. Ethics consultation services are typically expected to support a healthcare team by contributing to the adequate understanding, deliberation and/or the solution of an acutely occurring ethical dilemma or conflict situation. Decision-making usually takes place within a limited window of time, puts—besides the medical aspects of the case—respect for individual patient autonomy at the center of discussion, and can be viewed in general as a "firefighting" or "putting out fires" approach of doing ethics. Furthermore, and within the predominant biomedical model of care as well as treatment- and discharge-oriented processes of delivering care, individual patient autonomy shows itself to be the central concept “guaranteeing” that the patient's values become visible and are sufficiently integrated in the clinical decision-making process. Regardless of the diversity of approaches and methods in CEC, a narrow conception of autonomy predominates. Therein, "What does she want?" and "Has she the capacity to consent?" are the primary questions. In clinical practice, a focus on immediate problem-solving in the sense of rapidly eliciting patient’s wishes and preferences, proposing a plan of care, taking a joint (ethically principled) decision and—for the patient or patient’s representative—authorizing the healthcare professionals to perform health interventions (“informed consent”) prevails. In situations of crises where disease or injury suddenly or unexpectedly occur, where uncertainty dominates, or where there is high decisional pressure because of medical reasons, such a conceptualization of doing CEC has its justification and should not be put in a bad light. Nevertheless, one approach does not fit all circumstances. Particularly for the chronically ill, where everyday experience in dealing with symptoms and complex situations under lifeworld conditions enable more advanced coping mechanisms, the need for (sometimes recurring) hospital care is integrated into a different and longer journey of chronic illness. The situatedness of the chronic condition within a person’s lifeworld becomes particularly apparent when, in CEC, when discussion is about autonomy, autonomous choice, and the person’s will. Here, chronic illness allows for more depth regarding the person’s values, preferences, choices, and how and where they want to direct care.

In this article, we argue that a conceptualization of ethics consultation, having "firefighting" and immediate problem-solving at its center, being based both on a biomedical model of care and on a narrow conception of autonomy, does not do justice to patients with (severe) progressive chronic conditions and can lead to epistemic injustice. This is because a broader understanding of their experience of being chronically ill, how they develop ideas about their life, what they ascribe meaning to and, how chronic illness changes their personal and social identity needs to be more fully appreciated during the consultation. Contributing further to epistemic injustice is the significant influence in CEC services of the scientific epistemology, the logics, the language, and both the organizational and hierarchical structures of acute healthcare. Within this context and compared with the medical scientific aspects of a disease ("medical dimension"), experiences of illness ("existential dimension") are not given much attention (Greenhalgh et al., 2015; Thorne et al., 2000). This claim is supported by a plethora of patient’s accounts both in academic literature and popular press showing that the traditional model of care perceives illness experience with minor importance, such that patients are now calling on physicians to enlarge their view “to consider the illness in the context of a living person and move beyond a narrow focus on disease” (Lockerman, 2006, 31). Although, to our knowledge, there are no specific studies for CEC concerning this call, from our own experience as clinical ethicists working in the field for many years, we observe that in a culture, where illness experience is given little attention, there is a considerable risk that the patient’s voice is also curtailed in CEC. Within such a powerful environment as the acute-care medical system, professionals in clinical ethics are challenged to manage consultations under the constraints of clinical practice and with the forces of this specific social environment. In addition, that aspects of chronic illness experience receive little attention in "mainstream approaches of CEC" becomes apparent, for example, when taking a closer look at ethical decision-making models, which represent important instruments for clinical ethics and ethics consultation. The experiential dimensions of chronic illness are hardly considered in these models.

If one approach does not fit all circumstances, what would professionals in clinical ethics and CEC have to become aware of, in order make a step towards a comprehension of "clinical ethics practice" that might be more apt, particularly for (severe) chronic conditions (e.g., Chronic Obstructive Pulmonary Disease, End Stage Renal Disease, Parkinson’s Disease, Multiple Sclerosis, Chronic Fatigue Syndrome)? To answer this question, first, we will reflect on what is normatively important about chronic illness and thus must be addressed adequately in ethically difficult decision-making situations. Fundamental for this is an understanding of chronic illness experience and the extent to which illness experience contains normative elements. Second, using Miranda Fricker’s concept of epistemic injustice, we will set out how chronically ill patients experience epistemic injustice in healthcare in general. Third, we will look critically at clinical ethics and CEC, and we describe sources of epistemic injustice towards the chronically ill within this field. Finally, we will outline some ideas for improvement. Conceptually these ideas will be based on a broad understanding of CEC as a hermeneutical endeavor of openness and uncovering, where the meaning of what is morally salient becomes visible within the joint deliberative and interpretive process.

## What is Normatively Important About Chronic Illness?

 Chronic illness fundamentally alters a person’s life (Bury, 1982; Charmaz, 2000; Kleinman, 1989; Toombs, 2006; Williams, 2000). For those receiving a diagnosis of a (severe) chronic condition, a massive change in identity^1^—from being a healthy person to an ill person—can happen in a moment. Their familiar life and its taken-for-grantedness, their plans, and expectations for the future are suddenly overturned and they must learn a whole new way of living. Chronic conditions differ in terms of severity, complexity, and their impact on the person affected. Nonetheless, a number of studies in the social sciences (Locock & Ziébland, 2015) indicate that the onset of a chronic illness represents a rupture, a disruption in a person's life in many respects. As Michael Bury explains, “illness, and especially chronic illness, is precisely that kind of experience where the structures of everyday life and the forms of knowledge which underpin them are disrupted” (Bury, 1982, p. 169). Being chronically ill means that those affected are accompanied by the symptoms of the disease throughout life and usually must cope with continuous deterioration. The chronic condition’s lifelong duration and its progressive course are, in turn, associated with feelings of lack of control, uncertainty, unpredictability, and a fear of physical loss or further illness.

Because a person’s values and beliefs express themselves in their managing of and coping with a specific chronic condition, subjective illness experiences—a person’s experiences and the way of giving meaning to life, illness, and living with the illness—have normative significance in the sense that subjective illness experience^2^ shapes personal preferences, values, and norms with which the individual’s life plan is both preserved and adapted to this new condition. How someone encounters her "self" being altered by a non-curable disease that sometimes progresses in severity and how she incorporates the illness into her life story as well as the way of integration and redefinition of both personal and social identity, is the expression of what essentially constitutes this person and is therefore normatively relevant. Chronic illness, according to Charmaz, has a “profoundly moral cast” and “moral meanings arise through interaction in local worlds and specific lives” (2000, p. 277). Chronic illness “means much more than feeling physical distress, acknowledging symptoms, and needing care. It includes metaphor and meaning, moral judgments and ethical dilemmas, identity questions and reconstruction of self, daily struggles and persistent troubles” (Charmaz, 2000, p. 277). Moreover, Lucius-Hoene observes that the way a person relates to the changes, limitations, loss of abilities, and remaining life possibilities caused by illness strongly influences individual moral categories and often leads to a reflection of one’s own and others’ normative beliefs (Lucius-Hoene, 2002). Thus, how a person tackles the incurable, how she integrates the illness into her life narrative, how she relates to herself, to her body, to her environment, and how she forms her moral concepts in this complex process, must be adequately weighted in ethically difficult decision-making situations as encountered in clinical ethics consultation. This would do justice to the epistemic status of chronically ill people as knowers regarding important dimensions of their being ill.

## Epistemic Injustice in Healthcare

Miranda Fricker’s *epistemic injustice* is a concept at the nexus between epistemology, ethics, and politics (Fricker, 2007). It is the injustice done to a person's ability as an epistemic subject (i.e., as a subject who possesses knowledge, who can think and question) by not considering him or her an equal partner in conversation or negotiation. Typically, epistemic injustice occurs when a listener denies credibility or trustworthiness^3^ to a speaker. Epistemic injustice can express itself in manifold ways and to varying degrees and is influenced by a number of factors. Fricker identifies two wrongs that can be done to someone: *testimonial* injustice and *hermeneutical* injustice.

*Testimonial injustice* is grounded on a stereotyping assignment of an individual to a particular social group and occurs when prejudice causes a hearer to ascribe a deflated level of credibility to a speaker’s testimony (Fricker, 2007). In medical practice, a scientific epistemology and the biomedical model of disease are action guiding and go hand in hand with an understanding of the human body shaped by the natural sciences and a causal way of thinking (Sulik, 2011; von Uexküll & Wesiack, 2011). Not infrequently, this leads to non-scientific forms of knowledge being judged as unreliable. The medical experts' epistemic authority (i.e., the power of definition to decide what is considered as relevant, based on expertise, knowledge advantage, and professional experience), results in certain forms of evidence and knowledge being given greater weight. According to Carel, in healthcare, testimonial injustice can occur when health professionals simply ignore what is said and do not act on it, or listen, but dismiss what is said as irrelevant. Not only can epistemic injustice manifest itself in medical practice but also it can take the form of specific policies or even patient feedback collection forms (Carel, 2016).

Informational content typically communicated by a person with chronic illness that relates to existential dimensions of being ill (e.g., feelings of physical alienation, insecurity, or fear of social isolation) is judged to be of minor relevance for medical treatment. In general, such information receives little appreciation in acute medical health care and is mostly classified as "subjective" or "non-medical" and therefore—except when a problem is classified as "psycho-pathologically" relevant—usually gives little reason to initiate measures.

*Hermeneutic injustice* happens, according to Fricker, when certain social experiences that are exclusionary or problematic in nature lack the conceptualizations that could put these experiences into words. As an example, Fricker cites the experience of sexual harassment in a cultural context where this crucial concept is not (yet) present. Fricker calls this “a gap in collective hermeneutical resources” (Fricker, 2007, p. 7). When the necessary resources for understanding certain experiences are lacking, those experiences cannot be articulated or communicated to others. This in turn leads to hermeneutic marginalization, which she defines as “unequal hermeneutical participation with respect to some significant area(s) of social experience” (Fricker, 2007, p. 153). Hermeneutic injustice is always dependent on the circumstances and occurs within the context of particular social norms, practices, or institutional structures. Thus, chronically ill patients may experience hermeneutic injustice in healthcare settings focused on acute care because, for them, important experiences of illness are often not part of the "interactional repertoire" with those in charge of their medical treatment. Hermeneutic injustice or the "impossibility to convey" certain experiences is also accentuated by the fact that such experiences are difficult to access by others lacking them. Examples include experiencing childbirth, physical or psychological trauma, and extreme pain. It is similar with chronic illnesses. What it means for an affected person to have to live with a disease that, after diagnosis, will accompany her for the rest of her life is often difficult for people not so affected to understand.

Epistemic injustice is further exacerbated where healthcare systems are characterized by constant time pressure, shift work, many interfaces between different professionals involved in care, and routine procedures. In addition to highly formalized discussion structures (e.g., medical rounds or case discussions), where dialogue is often conducted in a technical jargon and according to strictly prescribed rituals, all these aspects contribute to the prevention of a culture in which conversational spaces would be available where patients could be listened to and their perspectives could be engaged with more deeply.

In view of the difficult epistemic status of chronically ill patients in acute care, CEC could assume responsibility to create and enable such spaces and opportunities. In doing so, clinical ethicists could take on the role of a "mediator" between the medical dimension of a disease and the existential dimension of illness. Doing so, CEC could contribute to counter the epistemic "indifference" in acute care and the ethics of crises by enabling the existential dimensions which are central to the reality of life for patients with chronic illness to be revealed and considered. To take on this role, however, clinical ethics would have to become aware of its own epistemic injustices and develop approaches that better suit existential aspects of being chronically ill.

## Sources of Epistemic Injustice in Clinical Ethics

As clinical ethics evolved within acute healthcare and having to establish itself within such a powerful and, even today, still strongly hierarchical context, it has been shaped by the rules and mechanisms of the medical system^4^ in many ways (Ho & Unger, 2015). Typically, when it comes to an ethics case consultation in clinical practice, the ethicist enters a complex situation with high moral tension, uncertainty, and a strong decisional pressure. At the center of deliberation are usually difficult decisions with serious consequences. Similar to the model of medical consultation (e.g., by a cardiologist, neurologist, or another medical specialist), CEC is commonly understood as a particular clinical service with the “central purpose to improve the process and outcomes of patient care by helping to identify, analyze, and resolve ethical problems” (Fletcher & Siegler, 1996, p. 125) and is conducted in a manner analogous to the solution of medical-scientific problems. As the process of ethical decision-making and problem solving is embedded in a clinical practice, which is characterized by a narrow scientific epistemology, non-scientific forms of knowledge and expression have little appreciation. Clinical ethics and CEC are not unaffected by this narrow epistemology, expressing itself also in terms of language. The influence of medical language on the language of ethicists was described in a 2013 essay by Canadian sociologist Arthur Frank. In that essay, Frank comments on a short notice in the *Hastings Center Report* about a visit in the late 1980s by a group of American bioethicists to what was then the Soviet Union: “At the core of the short notice was the observation that the Russian bioethics colleagues were struck by how fluent the American visitors were in medical jargon—how readily they shared physicians’ professional language. This medical fluency was presented as representing the advanced state of American bioethics” (Frank, 2013, p. 35). Ethicists’ medical fluency is reflected in that case reports or patient situations in CEC are often presented in a depersonalized, naturalistic manner analogous to a clinical case report. Much more emphasis is placed on medical measures, and it is not uncommon for the specific circumstances, the place, and those involved to be depicted rather superficially. Persons are referred to by initials or in their generic roles (e.g., the primary caregiver, the ward physician, or the patient or "pat".). Time is perceived in a specific way in that the description of relatively short clinical moment turns out to be extensive and detailed, while the much longer, non-clinical time spans are usually presented in a very abbreviated form—if at all. In clinical practice (and in ethics education in healthcare), ethical case presentations are to a large extent conventionalized, linguistic rituals. The vocabulary and discourse structure used entail implicit normative assumptions. In aligning the descriptions of patient situations in ethical case discussions to medical terminology, a reality is structured and constructed in which non-scientific perspectives on events, on the individual situation, the body, the suffering, or the experience of illness are either suppressed or of secondary importance. According to the German philosopher Theda Rehbock, in scientific terminology "the ethical" necessarily remains hidden. She claims that ethical questions and problems in medicine, which are also always associated with life phenomena such as pain, illness, suffering, or dying, can only be articulated in the medium of everyday language (Fiebach et al., 2016). Not only the language of ethicists is influenced by medical, scientific language but also the everyday language of patients who in the course of their illness develop medical fluency. Arthur Frank describes “the obligation of seeking of medical care as a ‘*narrative surrender'*” and marks it “as the central moment in modernist illness experience” (Frank, 1995, p. 6). According to Frank, “[t]he ill person not only agrees to follow physical regimens that are prescribed; she also agrees, tacitly but with no less implication, to tell her story in medical terms” (Frank, 1995, p. 6). By patients having to tell their story largely in medical terminology, in chronic illness, this "narrative surrender" leads to epistemic injustice. There is neither the appropriate space nor the vocabulary for the individual specifics of living and coping with such a disruptive experience as (severe) chronic illness, for so important experiential aspects as loss of control, grief over what has been lost, feelings of insecurity, fear of the future, or changes in relationships to unfold.

By adopting medical terminology, professionals in clinical ethics and CEC have not only moved away from the language of the patient, but have also distanced themselves from the existential, lifeworld dimensions of being ill. However, this should not lead to the conclusion clinical ethicists do not need to understand medical language. To some extent, ethicists must master medical language to facilitate communication in the clinical context, and to convey that they apprehend the medical-scientific dimensions and therapeutic implications that underlie a particular patient situation. Nevertheless, they must be aware of the limitations and of what is lost sight of in a discourse conducted in medical-scientific language, as well as of the implicit, normative attitudes in patient rounds, interdisciplinary case discussions, and documents such as discharge reports, information sheets or medical guidelines—and equally in ethics case discussions. Since, as Rehbock correctly observes, we create reality through language and with the language we use, we have power and influence, just as we have with our actions in general, for what language we use and how we express ourselves communicatively.

To enable understanding and to achieve epistemic justice, the appropriate linguistic tools must be available in clinical ethics decision-making. This is of particular importance in the context of chronic illness, where illness is not a temporary phenomenon. Structures of clinical ethics decision-making should counter epistemic injustice by incorporating—hermeneutically understood—concepts and terminology into the clinical ethics decision-making structure that enable the articulation of chronic illness experience. The person with chronic illness needs to be perceived as someone who has specific knowledge and unique experiences with her illness. Since the normative framework for deliberation is ultimately provided by the content and structures in ethical decision-making, the individual’s experience with chronic illness and her lifeworld must be appreciated elements within these structures. Accordingly, to uncover the moral outlook of patients and who and what has significance for them, clinical ethicists would have to broaden their standard methodological repertoire and apply methods that would be suitable to achieve this.

Another source of epistemic injustice in clinical ethics and CEC in the acute-care setting is its narrow conceptualization of patient autonomy. Socio-political and legal developments in Western societies have resulted in contemporary medical ethics and healthcare placing an emphasis on promoting individual patient autonomy. This is to ensure that treatment decisions are aligned with patients’ priorities and values and that they are taken freely and without undue influence from others. The normative and legal orientation towards the principle of respect for autonomy is reflected in many models of ethical decision-making and regulatory guidelines. Procedurally and legally, the respect for patient autonomy unfolds in that the "well-informed" patient—except in emergency situations—gives informed consent (or refusal) to a treatment plan. A prerequisite for the consent’s legal validity is that the person making the decision has both the decision-making capacity^5^ and legal authority to do so. If this is not the case, a third party is entrusted with decision-making.

Although autonomy is a central concept of ethical orientation in biomedical ethics, autonomy is not interpreted uniformly. Autonomy is commonly conceived as the patient's right to make decisions according to her own beliefs and values, independent of external coercion or influence. As an expression of individual liberty, our current understanding of patient autonomy was influenced by the British philosopher and economist John Stuart Mill. Following in part from Mill, the two American bioethicists Tom Beauchamp and James Childress, whose four principles approach has been dominant in medical ethics, emphasize individual freedom of action and non-interference by third parties in personal affairs. They characterize the autonomous individual as freely acting “in accordance with a self-chosen plan, analogous to the way an independent government manages its territories and sets its policies” and claim that “two general conditions are essential for autonomy: (1) *liberty* (independence from controlling influences) and (2) *agency* (capacity for intentional actions)" (Beauchamp & Childress, 2019, pp. 99–100).

In summary, freedom of action, freedom of choice, and freedom of decision are at the center of standard views of individual autonomy. In biomedical ethics, this focus on freedom is generally interpreted as a corrective response to paternalism, which gave patients little say. However, it is insufficient to conceive of autonomy primarily as a right of defense against paternalism and interference, equated with freedom of choice—particularly in chronic illness. Furthermore, autonomy reduced to shape short-term clinical decisions is insufficient for a model of care for chronic illness. Autonomy is not static or fixed. Rather, it must be understood as unfolding in a process. Resting upon his experience with long-term patients and within a contextualist framework, the American philosopher George Agich, for example, writes that “[a]utonomy as it concretely emerges in the practical world of everyday life as opposed to the ideal of theory, necessarily involves *processes of interpretation and negotiation*” (Agich, 1993, p. 87). Autonomy to be realized, requires a caring, understanding counterpart. Being human constitutively means being in relationships. What we determine ourselves to be is essentially tied to experiences we have had and is shaped by people we have met in the course of our lives and whom we are interdependent with in different ways. Particularly in (severe) chronic illness, where the person affected is both in an incessant process of grappling with a progressive illness that continuously changes life and in a vulnerable balance of multiple (inter)dependencies, autonomy needs to be understood as a complex, multi-layered process. This process cannot be decoupled from the experience of illness in the individual biographical, cultural, and social context. How someone forms her moral beliefs, how she negotiates within her life with illness, and how individual moral categories and ideas about living a good life are formed can neither be seen as a linear, non-contradictory process nor as a process that is ever finished. That little attention is paid to such complex processes in healthcare structures resting on acute medical care and a biomedical model of care is hardly surprising, but it needs to change.

In acute care and CEC, the epistemic authority of patients with chronic illness is further weakened in that there is a tendency to focus on single decisional moments in which the act of exercising autonomy is perceived as selective and self-contained. However, autonomy develops over time and cannot be understood as a single separate moment of choice between different options. Rather, autonomy is a dynamic process dependent on relational circumstances. Therefore, a conception of autonomy acknowledging the vulnerable bodily, psychologically, and socially balance of chronic illness and placing relational realities at its center, would be epistemically more appropriate for (severely) chronically ill. Moreover, for autonomy to be realized and to unfold, a caring ally is necessary. This aspect of care is particularly important in living with (severe) chronic illnesses, which due to their progression often require increasing support from others.

Helping professionals to appropriately respect personal autonomy will require, and in this we follow the American feminist philosopher Anne Donchin, “to *respond sensitively to the meaning illness* has for those in their care; to deploy their power and influence to restore and strengthen autonomy competencies; and support patients’ struggles to create new personal meanings out of the experience of disease, disorder or disability” (Donchin, 2000, p. 240, emphasis by the authors). For clinical ethicists (and other professionals being entrusted with existentially challenging, and often life-threatening, situations,) to counter epistemic injustice, respond to the meaning of illness, and support patients’ coping with illness, would require clinical ethics and CEC to move from an ethics of crisis toward an ethics of accompaniment, endurance, and care. Conceptualized in this way, clinical ethics could act as a ‘mediator’ and improve understanding between the different epistemic realities of healthcare professionals, patients (especially those with the kinds of chronic illness discussed above), and ethics experts themselves so that medical, existential, and moral dimensions of disease and illness would better merge into each other. For this to succeed, clinical ethics and CEC are called upon to expand their repertoire in terms of content, structure as well as methodological and communicative competencies. Furthermore, clinical ethics would have to rethink and specify its understanding of its own role in the context of the acute care setting in which immediate problem solving, a scientific epistemology, and the biomedical model of care prevail. This would imply that clinical ethics adopts an attitude of epistemic modesty—an attitude of “I know nothing”—which recognizes that epistemic knowledge resides with the chronically ill person. To make this knowledge visible, hermeneutically oriented approaches are needed, to help to explore and understand the patient’s and her social environment’s lifeworld knowledge of living with chronic illness.

## Conclusion

In practice, there are various conceivable ways in which clinical ethics could contribute to an expansion of the biomedical model of care and make chronic illness knowledge more visible. Clinical ethics consultation services are entrusted with prospective and retrospective case consultation and with ethics education. These well-established practices could be used to sensitize healthcare professionals to the transformed world of the chronically ill person, and to reflect on the shortcomings of the biomedical model of care and of an epistemology putting the natural sciences and medical language at its center. Moreover, methods and skills for the exploration of the patient’s lifeworld can be taught (as continuing professional development for ethics consultants and healthcare professionals). In our view, the repertoire should include narrative (Montello, 2020), hermeneutical (Carnevale, 2019; Porz et al., 2011), and phenomenological (Greenfield & Jensen, 2010) approaches. Further, retrospective case discussions and ethics education events could also be used to initiate a change in the perception of CEC to broaden the scope of its role beyond "firefighting" and crisis intervention. It should be conveyed that the process of CEC, in essence, is a hermeneutical endeavor of openness and uncovering, where the meaning of what is morally salient becomes visible within a joint deliberative and interpretive process, and where the elicitation of the patient’s perspective is essential.^6^

As for case consultation structures, based on a relational concept of autonomy and on an accompanying ethics of care, we think that the repertoire of opportunities to engage more deeply with the patients' lifeworld could be broadened in different ways and should extend over the entire continuum of care. In a processual, relational understanding, ethics would have the task of offering the chronically ill person and those close to them opportunities for ethical reflection throughout the entire process of illness. In "situations of crises" in the acute care setting, that require resolutions based on a valid understanding of the complexity of the situation, clinical ethics could contribute to an awareness of both the importance of epistemic modesty and the normative significance of illness experience, so that the (nonclinical) lifeworld of the chronic patient would be considered more adequately. To accomplish the latter, at the beginning of the hospitalization process identifying indicators (e.g., "red flags") could be introduced. Early in the treatment, such indicators (e.g., advanced chronic illness, limited capacity or inability to judge, communication deficits, necessity of support in daily life) could point to a patient's particular vulnerability and to possible ethically difficult situations that could arise in the course of treatment. When such indicators are present, the clinical ethicist would be contacted by the treatment team and would so be included in the care pathway. The ethicist’s early involvement would allow for more depth in engaging with the patients’ and those close to them. Particularly important in the context of chronic illness are informal caregivers, who ensure continuity beyond an acute event and give the chronically ill much support in many ways.

In addition to support during clinical phases, ethics could also gain importance in non-clinical periods. As part of an outpatient continuum, for example, regular "ethics consultation-hours" could be offered to patients and families. Within an "accompanying conceptualization of ethics" an ethicist would be an embedded member of the outpatient healthcare team who would be entrusted with maintaining the relationship with the chronically ill and their families, mediating between the medical and the existential dimension of illness. She would thereby delve into the patient's life story and help to identify normatively important aspects of a person’s lifeworld. Moreover, she would also give support in preparing for acute health crises by anticipating probable ethical dilemma situations within a specific illness trajectory.

Closure meetings could be another support service in the outpatient setting. Typically, after CEC in an acute medical event, for the clinical ethicist the relationship with the patient and her family ends with a consultation note being added to the medical record. There is also little systematic feedback from patients or their families about the impact of the ethics consultation (Cho et al., 2020; Mabel et al., 2020). To allow "closure" for those involved after ethically difficult and stressful decision-making situations and for the CEC, the introduction of a post-clinical meeting might be helpful, especially when aspects of the chronic illness experience continue to have ethical implications for managing their care. At present, such "closure" structures are mainly found in intensive care medicine, where decisions at the end of life are often accompanied by a high degree of psychological distress for those close to the patient. Schenker and Barnato, for example propose a “storytelling intervention” (Barnato et al., 2017). Particularly in the context of chronic illnesses, with their long history and a high degree of obligation towards the chronically ill, a storytelling intervention could contribute to a positive end of a chapter in a (life) story. Just as at hospital admission, indicators could be formulated for discharge planning to identify patients for whom periodic follow-up could be a supporting contribution to their outpatient care.

To be able to cover such a repertoire of teaching content and to take on a supportive role in care, the ethicist would need to acquire in-depth knowledge and understanding of chronic illness experience per se ("illness literacy") and specific knowledge regarding certain disease trajectories and be familiar with the most common medical problems associated with the disease ("disease literacy"). If the mediation between the existential, moral, and medical dimensions is to succeed, ethicists need not only a sound expertise in theoretical and applied medical ethics, but also hermeneutic (e.g., Porz et al., 2011), narrative (e.g., Montello, 2020), phenomenological (e.g., Greenfield & Jensen, 2010) competence and communicative skills—which in turn requires specific training.

We see several possible constraints and limitations. First, for the implementation of additional services of this kind, among healthcare professionals there is a need for awareness that the chronic patient’s illness experience is a crucial part of patient-oriented care. Beyond that and since healthcare professionals, especially physicians, would have to share power with ethicists, it would take a good working and trusting relationship between them. Second, budgetary constraints might be a major obstacle. Therefore, on both hospital management level and on the (political) level of the healthcare delivery system the necessary finances would have to be made available. Third, reflecting and coming to terms with the reality of one's own life and thinking about moral issues is a challenging process and could potentially be overwhelming for the chronically ill person and their families. It is therefore quite conceivable that they would rather avoid such conversations. Moreover, services of this kind might not meet some people’s needs. Finally, another difficulty could be that ethicists would not be comfortable with this accompanying role. Possibly they could be hesitant in taking on a role, in which they would have to delve much deeper into people's life stories, in which they would have to meet more expectations. Although this is likely to be associated with more responsibility on their part, it has the potential to enrich and sharpen the portfolio of clinical ethics by integrating the lived experience of people with chronic illness as a pivotal element of best practice in CEC.

## Footnotes

1 This claim does not apply to the same extent to chronic illness that develops slowly (e.g., diabetes or coronary heart disease).

2 By this we do not mean to say that only the experience of chronic illness shapes a person’s life. Other severely stressful life experiences (like the loss of a loved one, the experience of a natural disaster, or sexual abuse) and less serious events (like a divorce, the loss of a job etc.) have an influence of who we are, what we strive for, and what we give meaning to, to varying degrees.

3 This claim does not intend, however, to imply that speakers (patients) are always credible or trustworthy.

4 Of course, clinical ethics and CEC are not solely influenced by the medical system. Influencing factors also include, for example, the law, (moral) philosophy, and religious orientations in both a society and in healthcare institutions. These influencing factors will not be the subject of the considerations given here.

5 Decision-making capacity involves four cognitive abilities: The ability (1) to understand, (2) to communicate a clear choice, (3) to appreciate the situation and its consequences, and (4) to reason. People with chronic illness are particularly well positioned to appreciate their situation broadly – including how consequences of different options or decisions may play out in their lives. The situation for acute care decisions may not need to appreciate the situation and consequences as fully.

6 Granted, some CECs will respond to relatively straight forward questions of fact or interpretation about how a policy, codes of ethics, or other normative guidance, is applied in a specific case. Here we are drawing attention to roles beyond what is already well-established.
